# The Multi-Focus-Image-Fusion Method Based on Convolutional Neural Network and Sparse Representation

**DOI:** 10.3390/e23070827

**Published:** 2021-06-28

**Authors:** Bingzhe Wei, Xiangchu Feng, Kun Wang, Bian Gao

**Affiliations:** School of Mathematics and Statistics, Xidian University, Xi’an 710071, China; bingzhewei@126.com (B.W.); kwang96@stu.xidian.edu.cn (K.W.); gaobian0122@163.com (B.G.)

**Keywords:** multi-focus-image-fusion, sparse representation, convolutional neural network

## Abstract

Multi-focus-image-fusion is a crucial embranchment of image processing. Many methods have been developed from different perspectives to solve this problem. Among them, the sparse representation (SR)-based and convolutional neural network (CNN)-based fusion methods have been widely used. Fusing the source image patches, the SR-based model is essentially a local method with a nonlinear fusion rule. On the other hand, the direct mapping between the source images follows the decision map which is learned via CNN. The fusion is a global one with a linear fusion rule. Combining the advantages of the above two methods, a novel fusion method that applies CNN to assist SR is proposed for the purpose of gaining a fused image with more precise and abundant information. In the proposed method, source image patches were fused based on SR and the new weight obtained by CNN. Experimental results demonstrate that the proposed method clearly outperforms existing state-of-the-art methods in addition to SR and CNN in terms of both visual perception and objective evaluation metrics, and the computational complexity is greatly reduced. Experimental results demonstrate that the proposed method not only clearly outperforms the SR and CNN methods in terms of visual perception and objective evaluation indicators, but is also significantly better than other state-of-the-art methods since our computational complexity is greatly reduced.

## 1. Introduction

In the image processing field, multi-focus-image-fusion is a significant branch [1,2,3]. Multi-focus-image-fusion is the process of combining two or more images with different focal points of the same scene into a composite image with all-focus, which is of service to humans and machine perception [4,5]. The multi-focus-image-fusion holds true for multifarious applications such as remote sensing and computer vision [6].

In the past decade, sparse representation (SR)-based methods have been extensively applied to multi-focus-image-fusion [7]. SR has been proven as an extraordinarily powerful signal modeling method and has a good reputation in both theoretical research and practical application [8]. Yang and Li first applied SR in the field of image fusion [9]. After that, a large number of fusion methods based on SR emerged [10,11]. Liu and Wang proposed a adaptive sparse representation (ASR) model for simultaneous image fusion and denoising [12]. In the ASR model, a set of compact sub-dictionaries were learned from plentiful image patches which were pre-classified into several categories according to their gradient information. One of the sub-dictionaries can be adaptively selected by a given set of source image patches. In [13], a convolutional sparse representation (CSR)-based image fusion framework was presented, in which each source image is decomposed into a base layer and a detailed layer. The SR-based method was, in nature, the local one with the nonlinear fusion regulation, which was used to merge the source image patches.

In contrast to the relatively complex fusion method based on SR, in [14], Liu et al. proposed a multi-focus-image-fusion method based on the convolutional neural network (CNN). In this method, the decision map was received in line with the CNN model, which represented the accurate measurement of activity level. At last, a pixel-by-pixel weighted average strategy was employed to gain the fused image. Compared to SR methods, CNN was the global method with a linear fusion rule.

Uniting the merits of the two aforementioned methods, a novel fusion method was presented and more ample fused images were gained. In this method, source image patches are fused on the strength of the new weights obtained by CNN and SR.

The rest of this paper is organized as follows. In Section 2, some related work is discussed. In Section 3, the basic idea of the proposed fusion method is presented in detail. Experimental results and discussions are demonstrated in Section 4. Finally, Section 5 concludes the paper.

## 2. Related Works and Discussion

### 2.1. Sparse Representation

The ecumenical process of most SR-based methods is divided into three steps. Above all, the input images fall into a cluster of patches and the sparse codings of all patches are acquired [15]. Afterwards, the fused sparse vectors/codings are determined on account of a nonlinear fusion rule with the norm of the sparse vectors [16]. The final result was obtained by adopting reconstruction.

In the sparse coding step, SR handles the image patches by a pre-trained dictionary, ultimately gaining more concise representation [17,18,19,20]. Given a patch $s\in R^{n}$ and a trained dictionary $D=\left[d_{1},d_{2}\cdots d_{K}\right]\in R^{n\times K}\left(n<K\right)$ with atoms $d_{k}$, the SR of *s* was expressed as a sparse vector $x=[x_{1},x_{2},\cdots ,x_{K}]$ that did not merely meet s=Dx or s≈Dx, but also satisfied sparsity. This problem can be formulated as

(1) $$\underset{x}{\arg\min}{∥x∥}_{0},s.t.{∥s-Dx∥}_{2}<\varepsilon$$

where ${∥\cdot ∥}_{0}$ denotes a semi-norm that computes the number of nonzero entries in *x* and ε is the error tolerance. The $l_{0}$-minimization is a widespread NP-hard problem [21]. The approximation techniques cover greedy algorithms such as matching pursuit (MP) and orthogonal matching pursuit (OMP) that are extensively applied to resolve approximation matters [22,23]. *D* is a trained dictionary and is obtained via the K-singularly valuable decomposition (K-SVD) algorithm that is shown in Algorithm 1.

There are some issues that need further discussion. The sparse coding of each patch greatly increases the computational complexity. It is doubtful to what extent the magnitudes of the norm are consistent with the activity level of the corresponding patches. We therefore ask whether there is a better criterion that can be used to fuse the coefficients of SR.

### 2.2. CNN-Based Image Fusion Method

**Figure 1:**
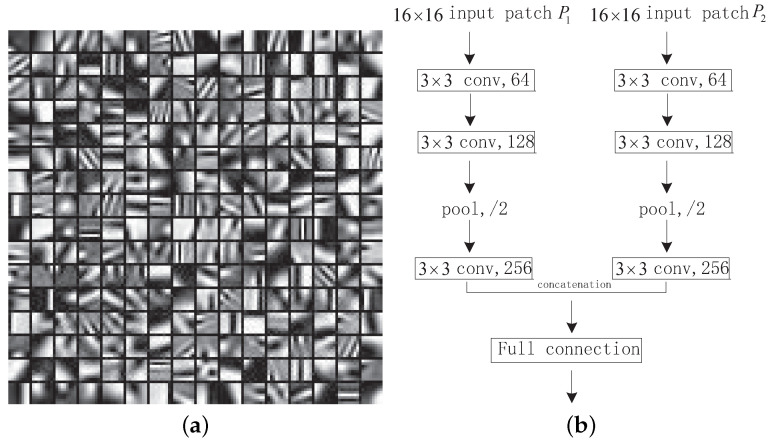
(**a**): The trained dictionary; and (**b**): the CNN model.

In [14], an emblematic CNN method for multi-focus-image-fusion is presented. Figure 1b is the CNN model used for fusion. It can be seen that each branch in the network has three convolutional layers and a max-pooling layer. The convolutional and max-pooling layers are considered as feature extraction. The output of Figure 1b is a 2-dimensional vector that is the two scores of the input image patches $P_{1},P_{2}$, which is fully connected with a 256-dimensional vector. The 2-dimensional vector produces a probability distribution on two classes. The fully connected layer can be deemed as classification. Then, the softmax loss function is applied to gain the value of the score map. Please note that in the fusion process, after the two fully connected layers are converted into convolutional layers, the network can process the source images of any size as a whole without dividing them into small patches [14]. The result of the CNN model is the score map that represents the pixels’ sharpness level. More particular information about the CNN model can be found in [14].
**Algorithm 1** Dictionary Learning (K-SVD)**Input:** Example $Y={\left\{y_{i}\right\}}_{i=1}^{N}$, initialize dictionary *D*, sparse matrix *X*,**Output:** Dictionary, sparse matrix.
1:**Initialize:** Randomly take *K* column vectors from the original sample $Y\in R^{m\times n}$ or take the first *K* column vectors $d_{1},d_{2},\cdots ,d_{K}$ of its left singular matrix as the atoms of the initial dictionary, and the dictionary $D_{0}\in R^{m\times K}$, j=0, maximum iterations *J*, tolerance value $\varepsilon_{0}$.2:**Sparse coding:** Using the dictionary $D_{j}$, $X_{j}\in R^{K\times n}$ is obtained by
(2)$$\min_{D,X}{∥Y-DX∥}_{F}^{2}s.t.\forall i,{∥x_{i}∥}_{0}\leq \varepsilon_{0}.$$3:**Dictionary update:** Update dictionary $D_{j}$ column by column, column $d_{k}\in \left\{d_{1},d_{2},\cdots ,d_{K}\right\}$.
When updating $d_{k}$, calculate the error matrix $E_{k}$, $E_{k}=Y-\sum_{j\neq k}d_{j}x_{T}^{j}$;Take out the set of indices where the *k*-th row vector $x_{T}^{k}$ of the sparse matrix is not 0, $\omega_{k}=\left\{i|1<i<n,x_{T}^{k}\left(i\right)\neq 0\right\}$, $x_{T}^{{}^{\prime }k}=\left\{x_{T}^{k}\left(i\right)|1<i<n,x_{T}^{k}\left(i\right)\neq 0\right\}$.Elect the column corresponding to $\omega_{k}\neq 0$ from $E_{k}$, and obtain $E_{k}^{{}^{\prime }}$.Perform singular value decomposition of $E_{k}^{{}^{\prime }}$, $E_{k}^{{}^{\prime }}=U\Sigma VT$, take the first column of *U* to update the *k*-th column of the dictionary, that is, $d_{k}=U\left(\cdot ,1\right)$; Let $x_{T}^{{}^{\prime }k}=\Sigma \left(1,1\right)V{\left(\cdot ,1\right)}^{T}$, after obtaining $x_{T}^{{}^{\prime }k}$, update accordingly it to the original $x_{T}^{k}$.Set j=j+1.4:**repeat**5:    The sparse coding and dictionary update steps;6:**until** the specified number of iteration steps *J* is reached, or converge to the specified error $\varepsilon_{0}$.

The specific steps of the CNN-based method are described below. Two source images are primarily sent to the CNN model to obtain a score map that includes the focus information of source images. Each pixel in the score map is acquired according to the focus characteristics of a pair of corresponding patches from the two source images. Consequently, the focus map with the size equal to that of source images is gained from the score map via averaging the overlapped patches. Afterwards, the focus map is divided into a binary map on the ground of a threshold of 0.5. Then, the binary map is optimized with a small region removal and guided image filtering to create the final decision map. At last, the fused image is obtained through the pixel-wise weighted-average algorithm.

There are still a few issues that require ulterior debate and need further discussion. The in-focused and out-focused regions of the source images are separated in the focus map. For the in-focused and out-focused junction area, the image patches are not well explained on the CNN model with a black box and it is easy to cause blockiness and artifacts. The CNN method employs the focus map to learn the decision map and the final fusion rule is linear. We therefore ask whether there is a better manner to utilize the map.

### 2.3. Complementary of the Two Methods

In accordance with the complementarities of SR and CNN, a novel multi-focus fusion method is proposed. In the first place, the weight map is acquired by means of the focus map obtained via the CNN model. Then, the source image patches obtained through the sliding window technique have strong correlation and spatial consistency. Meanwhile, these spatially adjacent patches have similar focusing conditions. If the patch is in-focused or out-focused, it can be directly drawn from the original images with any computation. At the junction of in-focused and out-focused areas, the new SR is employed. In the new SR, the weight norm is needed for measuring the activity level of the source image patches. The fused norm is obtained according to the magnitude of the weight norm. After that, the reconstruction is carried out to earn the fused image patches. Finally, the fused image is received through the pixel-wise weighted-average algorithm. To sum up, the multi-focus fusion method proposed in this paper gives each patch a suitable fusion rule.

The highlights of the mixed method based on SR and CNN include: (1) The sorting treatment of image patches based on the CNN model reduces the computational complexity of SR [24,25,26]; (2) The pixel value of the decision map obtained by means of the CNN model is imposed on the norm of sparse vectors, which can more accurately measure the activity level of the source image patches, giving full play to the advantages of strong spatial correlation between patches; (3) SR can handle the in-focused and out-focused junction areas that CNNs with black boxes cannot properly handle, making the patches in the junction area interpretable; and (4) SR can perform the nonlinear fusion of the patches at the junction of in-focused and out-focused area.

## 3. Proposed Fusion Algorithm

**Figure 2:**
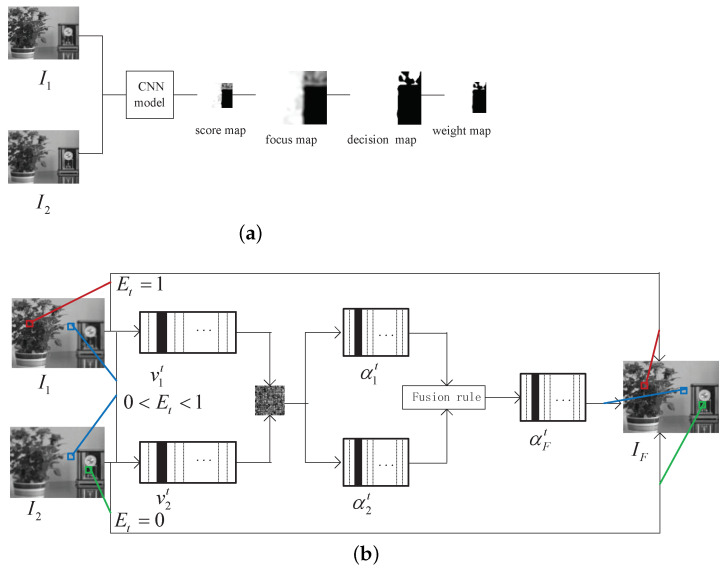
Flowchart of the proposed fusion algorithm: (**a**): Generation of weight map; (**b**): The process of the algorithm.

The proposed method based on CNN and SR includes three principal parts: (1) CNN-based weight map generation; (2) fusion of image patches based on the new SR; and (3) Fast image fusion based on patches. The following subsections describe the aforementioned steps at length. The algorithm flow is shown in Figure 2.

### 3.1. CNN-Based Weight Map Generation

We suppose that $I_{1},I_{2}$ are the two source images and the size is X×Y. $I_{1}$ is taken as a reference. These two images are fed to a pre-trained CNN to acquire the score map, the size of the which is [(X−16+2)/2]×[(Y−16+2)/2] (where [·] denotes the ceil operation). Every value of the score map that represents the focus level of a set of 16×16 patches of $I_{1}$ is between 0 and 1. The closer the pixel value is to 1, the more focused the image patches from $I_{1}$ are. After that, each pixel of the score map is extended to a 16×16 matrix that has the same element, and the focus map with the size X×Y is obtained through the pixel-wise overlap-averaging tactics. With this, the initial segment and small region removal were performed on the focus map to obtain the decision map. Later, the slider process is executed on the decision map. The patch size is 8×8 and the step size is 1. Each patch is averaged to obtain the pixel value of the corresponding position of the weight map *E*, i.e., the weight of the patches. The size of *E* is (X−8+1)×(Y−8+1). The flow chart for generating the weight map *E* is shown in Figure 2a.

### 3.2. Fusion of Image Patches Based on the New SR

Given the image patches $P_{q}$, q=1,2, which are represented as vectors $V_{q}$, the normalization is performed via $v_{q}=V_{q}-\bar{V_{q}}\cdot 1$, where $\bar{V_{q}}$ is the mean value of $V_{q}$.

Then, the normalized vectors $v_{q}$ are represented in the dictionary by the following formula:

(3) $$v_{q}=D\alpha_{q},\min{∥\alpha_{q}∥}_{0}$$

where *D* is the pre-trained dictionary via the K-SVD algorithm, as shown in Figure 1a. The $\alpha_{q}$ that are earned by the OMP algorithm are the SR vectors of $P_{q}$.

The fusion coefficients and the fusion means are, respectively, obtained as follows:

(4) $$\alpha_{F}=\left\{\begin{array}{c} \alpha_{1},ifM_{1}>M_{2}\\ \alpha_{2},otherwise \end{array}\right.$$

(5) $$\bar{V_{F}}=\left\{\begin{array}{c} \bar{V_{1}},ifM_{1}>M_{2}\\ \bar{V_{2}},otherwise \end{array}\right.$$

where $M_{1}=\omega \cdot {∥\alpha_{1}∥}_{1}$, $M_{2}=\left(1-\omega \right)\cdot {∥\alpha_{2}∥}_{1}$ and ω is the weight of $P_{1}$ obtained from *E*. The weight $l_{1}$-norm $M_{q}$ reflects the actual activity level of the image patches, which can avoid the wrong selection of patch with the small value of the norm.

The fused result of $V_{F}$ is calculated by

(6) $$V_{F}=D\alpha_{F}+\bar{V_{F}}$$

$V_{F}$ is reshaped into the 8×8 patch $P_{F}$ and $P_{F}$ is the fused image patch. In the end, each pixel’s value of the fused image $I_{F}$ is obtained by its average over its superposition.

### 3.3. Fast Image Fusion Based on Patches

By sliding window technology, $I_{1},I_{2}$ is divided into n×n patches $I_{1}^{t},I_{2}^{t}$, t=1,⋯,T. The number of patches from each image is *T*, T=(X−n+1)(Y−n+1). In fact, the procedure proposed in Section 3.2 is not needed for each patch. In the very beginning, the weight map *E* is expressed in vector form $E_{t}$ that is used to choose the patch that does not need sparse coding.

**Figure 3:**
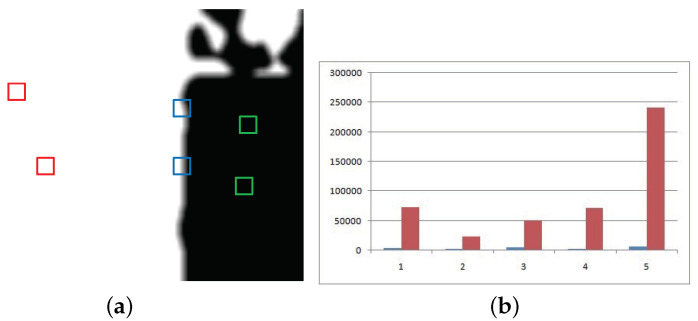
(**a**): The weight map; and (**b**): The number comparison of positions of image patches.

When $E_{t}=1$, i.e., the image patch of $I_{1}$ is in-focused, as can be shown, for example, when these in-focused patches are at the position of the red diamonds in Figure 3a, then the fusion result is $I_{F}^{t}=I_{1}^{t}$.

If $E_{t}=0$, i.e., the image patch of $I_{1}$ is out-focused, as shown by the green squares in Figure 3a, the fusion result $I_{F}^{t}$ is $I_{2}^{t}$.

In the case of $0<E_{t}<1$, the image patch is located somewhere in between the in-focused and out-focused regions. For instance, these patches are the blue blocks in Figure 3a. Only in this case is the new SR fusion method adopted, where $\omega =E_{t}$ and the fusion patch is gained.

It can be known that the above classification can greatly reduce the computational complexity.

## 4. Experiments

This section successively gives the experimental settings that include the source images to be processed, image fusion quality metrics, parameters setting, computational complexity analysis, compared methods and image fusion results to be visually and quantitatively analyzed.

### 4.1. Source Images

**Figure 4:**
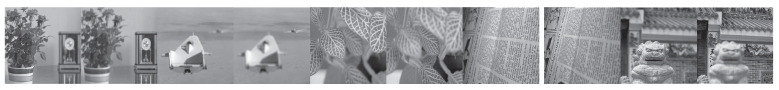
The multi-focus grayscale source images.

**Figure 5:**
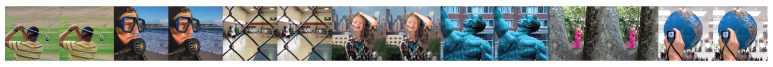
The multi-focus color source images.

In order to illustrate the experimental results, different types of source images are applied. There are 12 pairs of source images, including five pairs of multi-focus grayscale images in Figure 4 and seven pairs of multi-focus color images in Figure 5. These images are obtained from the Lytro Multi-Focus Dataset that contains 20 pairs of color multi-focus images and four series of multi-focus images with three sources, and the Multi-Focus-Image-Fusion-Dataset that includes 150 different images used in multi-focus-image-fusion algorithms [27,28].

### 4.2. Evaluation Metrics

For the sake of verifying the performance of image fusion methods, subjective and objective evaluation metrics are usually applied. Between them, subjective evaluation means that people explain the relative merits of the methods through the visual effects of the fusion results, and it is affected by uncertain factors such as the observer’s own conditions, professional knowledge, observation angle, application occasions and objective environment [29]. The subjective evaluation is thus less reliable and objective. The objective evaluation is required to assist subjective evaluation. Therefore, the objective evaluation is especially important. The objective evaluation method conducts the quantitative analysis of fused images through certain mathematical models, which can overcome the limitations of subjective evaluation and the evaluation results are stable and reliable [30]. Generally speaking, it is difficult to objectively evaluate the merit and fallacies of the fusion method by relying on only one evaluation index. Therefore, many researchers ecumenically adopt comprehensive evaluation with multiple evaluation indexes.

In this paper, five metrics were employed to evaluate the fusion quality. The larger the values of the metrics, the higher the fusion performance. The five metrics are introduced as follows:

1. Mutual information mainly reflects how much information the fused image contains from the source images [31]. The greater the mutual information is, the more information of the source images the fused image contains, and the better the fusion effect is. Mutual information is defined as follows:
  (7) $$MI=MI\left(I_{1},I_{F}\right)+MI\left(I_{2},I_{F}\right)$$
  (8) $$MI\left(I_{q},I_{F}\right)=\sum_{x,y}h_{I_{q},I_{F}}\left(x,y\right)\log_{2}\frac{h_{I_{q},I_{F}}\left(x,y\right)}{h_{I_{q}}\left(x\right)h_{I_{F}}\left(y\right)}$$
  Here, $h_{I_{q}}\left(x\right),h_{I_{F}}\left(y\right)$ are, respectively, the edge histogram of $I_{q},I_{F}$. $h_{I_{q},I_{F}}\left(x,y\right)$ are normalized joint histograms of $I_{F}$ and source images $I_{q}$, respectively.
2. The Chen–Blum metric $Q_{CB}$ is a human perception-inspired fusion metric. $Q_{CB}$ is calculated by the following steps.
  At the very start, the masked contrast map for the input image $I_{q}\left(x,y\right)$ can be computed in:
  (9) $$C_{I_{q}}^{\prime }=\frac{l{\left(C_{I_{q}}\right)}^{n}}{k{\left(C_{A}\right)}^{p}+m}$$
  where *C* is Peli’s contrast, k,l,m,n are real scalar parameters, and more details on the parameter settings can be found in [32].
  The information preservation value $Q_{I_{q},I_{F}}\left(x,y\right)$ and the saliency map $\mu_{I_{q}}\left(x,y\right)$ can be calculated by the two following expressions:
  (10) $$Q_{I_{q},I_{F}}\left(x,y\right)=\left\{\begin{array}{c} {C^{\prime }}_{I_{q}}\left(x,y\right)/{C^{\prime }}_{I_{F}}\left(x,y\right),if{C^{\prime }}_{I_{q}}\left(x,y\right)<{C^{\prime }}_{I_{F}}\left(x,y\right)\\ {C^{\prime }}_{I_{F}}\left(x,y\right)/{C^{\prime }}_{I_{q}}\left(x,y\right),otherwise \end{array}\right.$$
  (11) $$\mu_{I_{q}}\left(x,y\right)=\frac{C_{I_{q}}^{\prime }\left(x,y\right)}{C_{I_{1}}^{\prime }\left(x,y\right)+C_{I_{2}}^{\prime }\left(x,y\right)}$$
  Then, the value of the global quality map can be calculated:
  (12) $$Q_{GQM}=\sum_{x,y}\mu_{I_{1}}\left(x,y\right)Q_{I_{1},I_{F}}\left(x,y\right)+\mu_{I_{2}}\left(x,y\right)Q_{I_{2},I_{F}}\left(x,y\right)$$
  where $Q_{CB}$ is the average of $Q_{GQM}$.
3. The fusion metric $Q_{G}$ based on the gradient is a popular fusion metric which computes the amount of gradient information of the source images injected into the fused image [33]. It is calculated by
  (13) $$\begin{array}{c} Q_{G}=\frac{\sum_{x=1}^{X}\sum_{y=1}^{Y}\left(Q_{e}^{I_{1},I_{F}}\left(x,y\right)Q_{o}^{I_{1},I_{F}}\left(x,y\right)\tau^{I_{1}}\left(x,y\right)+Q_{e}^{I_{2},I_{F}}\left(x,y\right)Q_{o}^{I_{2},I_{F}}\left(x,y\right)\tau^{I_{2}}\left(x,y\right)\right)}{\sum_{x=1}^{X}\sum_{y=1}^{Y}\left(\tau^{I_{1}}\left(x,y\right)+\tau^{I_{2}}\left(x,y\right)\right)} \end{array}$$
  where $Q_{e}^{I_{q},I_{F}}\left(x,y\right)$ and $Q_{o}^{I_{q},I_{F}}\left(x,y\right)$ are the edge strength and orientation reservation values, respectively. The weight factor $\tau^{I_{q}}\left(x,y\right)$ shows the significance of $Q^{I_{q},I_{F}}\left(x,y\right)$.
4. The fusion metric based on phase congruency $Q_{P}$ measures the image-salient features of the source images, such as the edges and corners in the fused image [34]. The definition of $Q_{P}$ is:
  (14) $$Q_{P}={\left(R_{r}\right)}^{\theta }{\left(R_{H}\right)}^{\upsilon }{\left(R_{h}\right)}^{\sigma }$$
  where r,H,h refer to phase congruency, maximum and minimum moments, respectively. The exponential parameters θ,υ,σ are all set to 1. More details about $Q_{P}$ can be seen in [34].
5. $Q_{Y}$ was proposed by Yang et al., which is a structural similarity-based method of fusion assessment [35]. The definition of $Q_{Y}$ is shown as follows:
  (15) $$Q_{Y}=\left\{\begin{array}{c} \max\left\{SSIM\left(I_{1},I_{F}\left|\omega \right.\right),SSIM\left(I_{1},I_{F}\left|\omega \right.\right)\right\},ifSSIM\left(I_{1},I_{2}\left|\omega \right.\right)<0.75\\ \mu \left(\omega \right)SSIM\left(I_{1},I_{F}\left|\omega \right.\right)+\left(1-\mu \left(\omega \right)\right)SSIM\left(I_{2},I_{F}\left|\omega \right.\right),otherwise \end{array}\right.$$
  The details of local weight μ(ω) and the structural similarity of images $SSIM(I_{1},I_{2})$ can be found in [35,36].

### 4.3. Parameters Setting

In this section, our training parameters are set. For image processing applications based on SR, the size of image patches is 8×8 and the step length of sliding window technology is 1 pixel, which has been proven to be an appropriate setting [37]. The dictionary is obtained according to the K-SVD methodology, and the 68,000 image patches are randomly selected from the natural images. According to the paper [38], NSCT is selected for the multi-focus-image-fusion-based MST and MST-SR methods. The implementation of the compared method in this article was based on the exposed code, and we set the parameters according to their original reports. All experiments were performed on MATLAB R2017a. The computer processor is Intel(R) Xeon(R) Silver 4110CPU.

### 4.4. The Compared Methods

The effectiveness of the proposed algorithm was evaluated against state-of-the-art research methods. The first one was the NSCT-based method that uses the weighted average for low-pass sub-bands and ’max-absolute’ for high-pass sub-bands. The second compared algorithm was based on SR [9]. The third method was ASR [12]. The fourth method was NSCT-SR-1 [38]. Each of the pre-registered source images was decomposed by NSCT 1 level decomposition, and the low-pass and high-pass coefficients were obtained. The low-pass coefficients were merged with an SR-based fusion method, while the high-pass coefficients were fused using the absolute values of coefficients for activity level measurement. The fifth approach was CSR [13]. The sixth compared algorithm was based on CNN [14].

### 4.5. Computational Complexity Analysis

In order to verify that the proposed algorithm reduced the computational complexity, Figure 3b is given. As shown in Figure 3b, the ordinate is the number of positions, and the abscissas 1–5 indicate five pairs of grayscale source images. The red and blue rectangles indicate the corresponding positions of the patches fused by SR in the traditional SR and the algorithm proposed in this paper, respectively. It can be seen from the histogram that the height of the yellow rectangle is much higher than that of the purple. Therefore, the fusion algorithm proposed in this paper greatly reduces the number of patches that need to be fused by SR, thereby enormously reducing the computational complexity.

Referring to Table 1, it can be seen that the running time of CNN-SR is less than that of the SR and CNN. The bold font in Table 1 indicates that the shortened running time of CNN-SR is greater than one minute. To sum up, the method proposed in this paper improves the computational efficiency.

### 4.6. Validity of the Proposed Fusion Method

**Figure 6:**
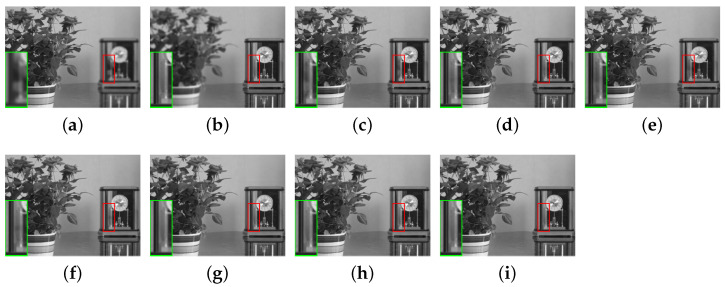
The ‘flowerpot’ source images and fusion results obtained by different fusion methods: (**a**) source image flowerpot A; (**b**) source image flowerpot B; (**c**) NSCT; (**d**) SR; (**e**) ASR; (**f**) NSCT-SR-1; (**g**) CSR; (**h**) CNN; and (**i**) CNN-SR.

**Figure 7:**
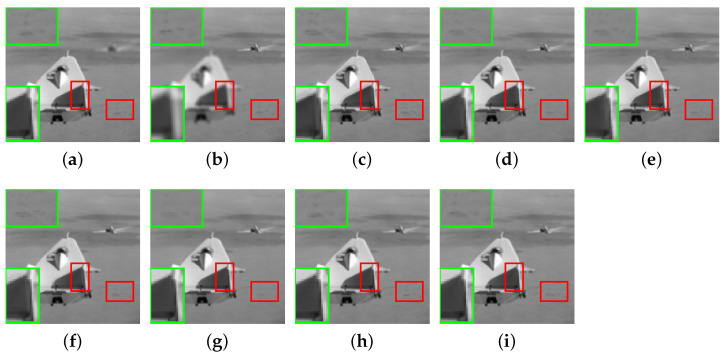
The ‘aircraft’ source images and fusion results obtained by different fusion methods: (**a**) source image aircraft A; (**b**) source image aircraft B; (**c**) NSCT; (**d**) SR; (**e**) ASR; (**f**) NSCT-SR-1; (**g**) CSR; (**h**) CNN; and (**i**) CNN-SR.

**Figure 8:**
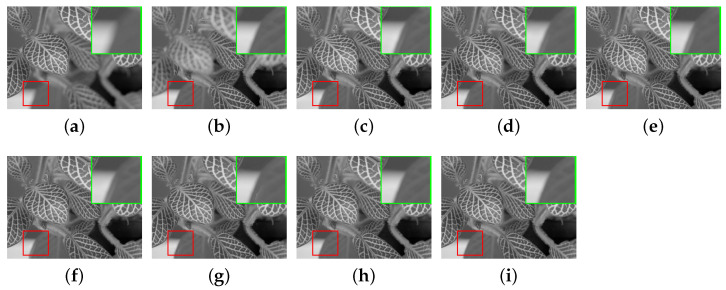
The ‘leaf’ source images and fusion results obtained by different fusion methods: (**a**) source image leaf A; (**b**) source image leaf B; (**c**) NSCT; (**d**) SR; (**e**) ASR; (**f**) NSCT-SR-1; (**g**) CSR; (**h**) CNN; and (**i**) CNN-SR.

**Figure 9:**
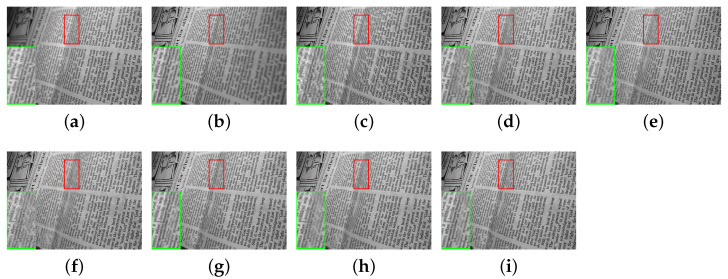
The ‘newspaper’ source images and fusion results obtained by different fusion methods: (**a**) source image newspaper A; (**b**) source image newspaper B; (**c**) NSCT; (**d**) SR; (**e**) ASR; (**f**) NSCT-SR-1; (**g**) CSR; (**h**) CNN; and (**i**) CNN-SR.

**Figure 10:**
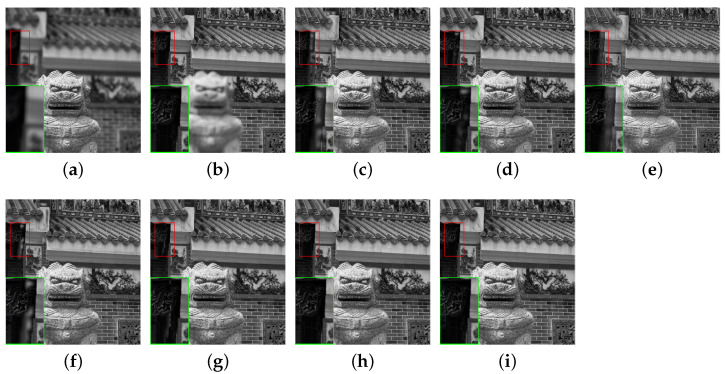
The ‘temple’ source images and fusion results obtained by different fusion methods: (**a**) source image temple A; (**b**) source image temple B; (**c**) NSCT; (**d**) SR; (**e**) ASR; (**f**) NSCT-SR-1; (**g**) CSR; (**h**) CNN; and (**i**) CNN-SR.

In this section, the comparative methods and the proposed method are applied to the commonly used multi-focus grayscale images of Figure 4. Figure 6a,b, Figure 7a,b, Figure 8a,b, Figure 9a,b and Figure 10a,b are source images from the same sensor, that are focused at different locations. The objects in other positions are out-focused and blurred. Figure 6c–i,Figure 7c–i, Figure 8c–i, Figure 9c–i and Figure 10c–i are the fusion results of different methods that are, respectively, MST(NSCT), SR, ASR, MST-SR(NSCT-SR-1), CSR, CNN and the fusion method CNN-SR proposed in this paper. The evaluation indexes are shown in Table 2, Table 3, Table 4, Table 5 and Table 6, and the values for the best fusion performance are bolded.

An example of ’flowerpot’ fusion is shown in Figure 6. The left-hand magnification of the clock is shown at the lower left corner of each image in Figure 6. From the magnified details, it can be seen that the fusion results obtained by MST, SR, ASR and MST-SR are uneven in varying degrees. For the remaining three fusion results, the human visual system struggles to tell the difference. Hence, objective evaluation is needed.

As shown in Table 2, among the five indicators, CNN-SR leads four, including $MI,Q_{G},Q_{Y},Q_{P}$. It follows that our proposed method can extract the most information from the source images. Although the $Q_{CB}$ of our proposed method is a little small, our proposed fusion method best preserves the structure and detailed information of the source images, and improves the clarity of the fused image.

Figure 7 shows the fusion results of the ‘aircraft’ images. In megascopic details, in the top left corner, the artificial information increases in Figure 7c,e. The fusion result based on SR Figure 7d has a slight artificial edge; the details of Figure 7f are inconspicuous; the details of Figure 7g,h are missing. In the enlarged area of the bottom left corner, there are different degrees of unevenness in Figure 7c,d,f. The fusion result of the algorithm in this paper shown in Figure 7i retains the best and restores the information at the bottom left.

Table 3 exhibits the objective evaluation of Figure 7. The objective results confirm that our approach is the best among the seven methods. The performance shows that the CNN-SR can extract the edge and structure information of the source image well.

As can be seen from the magnified details of Figure 8, the edge artifacts exist in Figure 8c–f, and the fusion results have low contrast, resulting in the loss of some useful details. Figure 8g with artificial edges is derived by the CSR-based approach. Figure 8h,i effectively preserve the detail of the source images without producing specific visual artifacts and brightness distortion. By comparison, using our method can achieve better image appearance.

The fusion performance measured by the objective metrics is shown in Table 4. The fusion method proposed in this paper is superior to other methods in terms of evaluation criteria $MI,Q_{CB},Q_{G},Q_{Y}$. The performance of these indexes shows that the fused image obtained by the CNN-SR method not only contains more detailed information, however, it is also more suitable for human visual perception. While the $Q_{P}$ is inferior to the CNN-based approach, our approach obtains comparable performance. Therefore, the proposed fusion method is superior to the SR-based method.

The fused results of ‘newspaper’ are shown in Figure 9. The fusion details are shown in the lower left corner of all the images in Figure 9. By comparing the image details fused by different methods, it can be determined that Figure 9c–g are relatively fuzzy, with poor contrast brightness. The fused images Figure 9h,i have better performance in information recovery and contrast and have better fusion performance.

The indicators of the proposed method and the contrastive methods are shown in Table 5. Table 5 shows the best performance of our proposed fusion method on $MI,Q_{CB},Q_{G},Q_{Y}$. It can be inferred that the proposed method has better performance in visual fidelity, image clarity and structure information level. For $Q_{P}$, the image integrated by CSR algorithm shows the best result. However, the image integrated by CSR extracts less information than the image fused by our proposed method. Therefore, the fusion method proposed in this paper is superior to the other contradistinctive methods.

The image pairs ‘temple’ and fusion results are shown in Figure 10. The details are shown in the lower left corner of Figure 10. For the details in Figure 10, the integrated images of MST, SR, ASR, MST-SR and CSR have different degrees of artifacts. The fused image of CNN and our proposed method show better performance in terms of detailed information than other integrated images.

The objective evaluation indexes are listed in Table 6. It can be clearly seen in Table 6 that our method obviously obtains all the largest quality indicators. A conclusion can be drawn from the experiment. Through visual comparison and objective evaluations, the proposed method shows emulatory fusion performance compared with the previous methods.

Therefore, these experimental consequences show that the proposed method fully extracts the information of the multi-focus source images. After CNN-SR fusion, the fused images with clear edges and no artificial artifacts preserve the detailed information well and have high contrast. Meanwhile, uneven fusion does not occur. Both the subjective and objective evaluation of CNN-SR are better than that of other algorithms.

### 4.7. Fusion of Multi-Focus Color Images

**Figure 11:**
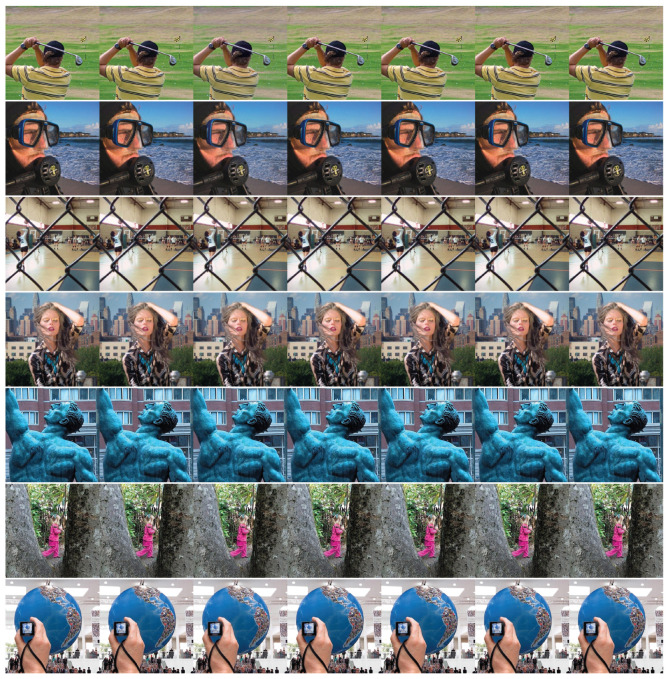
From left to right are the results of multi-focus color source images of MST, SR, ASR, MST-SR, CSR, CNN and CNN-SR.

The proposed method can be extended to multi-focus color image fusion. In order to prove the effectiveness of CNN-SR in the color images, the color source images, as shown in Figure 5, are adopted. Figure 11 is the fusion results of the different methods. Table 7 provides the average scores of the seven pairs of input images under different fusion methods. The visual fusion results and the quantitative estimate in Table 7 show that the CNN-SR method can gain the best fusion results.

## 5. Conclusions

We proposed a multi-focus-image-fusion method based on CNN and SR. In the method, the weight map was acquired according to the CNN model, where each pixel of the weight map represents the focus level of each source image patch. If the pixel value of the weight map is 0 or 1, this means that the image patch is in-focused or out-focused, which can be directly obtained from the source images. When the pixel value is greater than 0 and less than 1, it indicates that the image patch is between clear and blurred. The new SR method was adopted. In the novel SR method, the image patches are represented by the dictionary to gain the sparse vectors, and the weight of the patch is multiplied by the $l_{1}$-norm of its sparse vector to obtain its actual activity level. The fused sparse vectors were received by the max weight $l_{1}$-norm. The fused image can be gained by aggregating all the reconstructed patches with the pixel-wise overlap-averaging tactics. The classified disposal of the image patches makes the proposed fusion method have great computational efficiency and it retains as much information of source images in the fused image as possible. The qualitative and quantitative comparisons show that the proposed method achieves better fusion performance in visual and objective evaluation.

## Figures and Tables

**Table 1:** Running time with the units of the five grayscale images under different fusion methods.

Image	Flowerpot	Aircraft	Leaf	Newspaper	Temple
SR	**165.6602**	51.6356	118.1120	**168.7816**	**578.9371**
CNN	143.2177	68.1374	125.2807	148.8524	319.9401
CNN-SR	**105.2181**	47.8837	91.1454	**102.0202**	**265.0994**

**Table 2:** Objective evaluations of image pair ‘flowerpot’ fusion experimentations.

Metric	MST	SR	ASR	MST-SR	CSR	CNN	CNN-SR
MI	0.9463	1.1033	0.9587	1.0777	0.9837	1.1656	**1.1754**
$Q_{CB}$	0.7083	0.7256	0.7285	0.7332	0.7149	**0.7490**	0.7463
$Q_{G}$	0.6118	0.6116	0.6123	0.6225	0.5492	0.6461	**0.6480**
$Q_{p}$	0.8996	0.9220	0.9117	0.9207	0.9164	**0.9239**	**0.9239**
$Q_{Y}$	0.9490	0.9469	0.9508	0.9539	0.8837	0.9728	**0.9756**

**Table 3:** Objective evaluations of image pair ‘aircraft’ fusion experimentations.

Metric	MST	SR	ASR	MST-SR	CSR	CNN	CNN-SR
MI	1.1138	1.2690	1.1585	1.2436	1.1774	1.3457	**1.3575**
$Q_{CB}$	0.7115	0.7182	0.6855	0.7202	0.6692	0.7547	**0.7596**
$Q_{G}$	0.5990	0.6237	0.5975	0.6213	0.4985	0.6670	**0.6727**
$Q_{p}$	0.7522	0.7627	0.7580	0.7643	0.7062	0.7882	**0.7915**
$Q_{Y}$	0.9089	0.9294	0.9154	0.9217	0.8515	0.9728	**0.9777**

**Table 4:** Objective evaluations of image pair ‘leaf’ fusion experimentations.

Metric	MST	SR	ASR	MST-SR	CSR	CNN	CNN-SR
MI	0.6697	0.9286	0.6878	0.9285	0.8012	0.9055	**1.0012**
$Q_{CB}$	0.7463	0.7691	0.7249	0.7777	0.7747	0.7812	**0.7905**
$Q_{G}$	0.6527	0.6648	0.6561	0.6744	0.6381	0.6822	**0.6871**
$Q_{p}$	0.8121	0.8360	0.8207	0.8360	0.8307	**0.8456**	0.8409
$Q_{Y}$	0.9569	0.9660	0.9601	0.9703	0.9480	0.9845	**0.9894**

**Table 5:** Objective evaluations of image pair ‘newspaper’ fusion experimentations.

Metric	MST	SR	ASR	MST-SR	CSR	CNN	CNN-SR
MI	0.2916	0.7365	0.3290	0.6094	0.5955	0.7975	**0.8579**
$Q_{CB}$	0.6639	0.7282	0.6865	0.7094	0.7337	0.7403	**0.7462**
$Q_{G}$	0.5876	0.6332	0.6142	0.6150	0.6342	0.6434	**0.6513**
$Q_{p}$	0.4900	0.6256	0.5959	0.6012	**0.6459**	0.6449	0.6453
$Q_{Y}$	0.9371	0.9781	0.9677	0.9586	0.9854	0.9866	**0.9932**

**Table 6:** Objective evaluations of image pair ‘temple’ fusion experimentations.

Metric	MST	SR	ASR	MST-SR	CSR	CNN	CNN-SR
MI	0.4136	0.8377	0.4172	0.8163	0.7003	0.8977	**0.9388**
$Q_{CB}$	0.6809	0.7845	0.6421	0.7739	0.7928	0.8064	**0.8102**
$Q_{G}$	0.6510	0.7045	0.6452	0.7077	0.6901	0.7168	**0.7197**
$Q_{p}$	0.6428	0.7840	0.6577	0.7653	0.7734	0.7920	**0.7947**
$Q_{Y}$	0.9356	0.9756	0.9355	0.9675	0.9742	0.9928	**0.9951**

**Table 7:** Quantitative assessments of Figure 11 and values for the seven pairs of input images in Figure 11 are averaged.

Metric	MST	SR	ASR	MST-SR	CSR	CNN	CNN-SR
MI	0.9237	1.0558	0.9219	1.0900	0.9601	1.1105	**1.1459**
$Q_{CB}$	0.7629	0.7845	0.7426	0.8009	0.7758	0.8105	**0.8142**
$Q_{G}$	0.6918	0.7036	0.6979	0.7131	0.6547	0.7187	**0.7209**
$Q_{p}$	0.8326	0.8329	0.8322	0.8447	0.8327	**0.8478**	0.8471
$Q_{Y}$	0.9663	0.9730	0.9699	0.9776	0.9444	0.9847	**0.9869**

## Data Availability

Publicly available datasets were analyzed in this study: Lytro Multi-Focus Dataset: Mansour Nejati. 2015. https://mansournejati.ece.iut.ac.ir/content/lytro-multi-focus-dataset, accessed on 7 December 2020; Multi-Focus-Image-Fusion-Dataset: 2018. https://github.com/sametaymaz/Multi-focus-Image-Fusion-Dataset, accessed on 7 December 2020.

## References

[1] Meher B., Agrawal S., Panda R., Abraham A. (2019). A survey on region based image fusion methods. Inf. Fusion.

[2] Guo L.L., Woźniak M. (2021). An image super-resolution reconstruction method with single frame character based on wavelet neural network in internet of things. Mob. Netw. Appl..

[3] Woźniak M., Polap D. (2020). Soft trees with neural components as image-processing technique for archeological excavations. Pers. Ubiquitous Comput..

[4] Liu Y., Wang L., Cheng J., Li C., Chen X. (2020). Multi-focus image fusion: A survey of the state of the art. Inf. Fusion.

[5] Farid M.S., Mahmood A., Al-Maadeed S.A. (2019). Multi-focus image fusion using content adaptive blurring. Inf. Fusion.

[6] Panigrahy C., Seal A., Mahato N.K. (2020). Fractal dimension based parameter adaptive dual channel PCNN for multi-focus image fusion. Opt. Lasers Eng..

[7] Nejati M., Samavi S., Shirani S. (2015). Multi-focus image fusion using dictionary-based sparse representation. Inf. Fusion.

[8] Wang K.P., Qi G.Q., Zhu Z.Q., Chai Y. (2017). A novel geometric dictionary construction approach for sparse representation based image fusion. Entropy.

[9] Yang B., Li S.T. (2009). Multifocus image fusion and restoration with sparse representation. IEEE Trans. Instrum. Meas..

[10] Li Y.Y., Sun Y.J., Huang X.H., Qi G.Q., Zheng M.Y., Zhu Z.Q. (2018). An image fusion method based on sparse representation and sum modified-Laplacian in NSCT domain. Entropy.

[11] Li S.T., Yin H.T., Fang L.Y. (2012). Group-sparse representation with dictionary learning for medical image denoising and fusion. IEEE Trans. Biomed. Eng..

[12] Liu Y., Wang Z.F. (2014). Simultaneous image fusion and denoising with adaptive sparse representation. IET Image Process..

[13] Liu Y., Chen X., Ward R.K., Wang Z.J. (2016). Image Fusion with Convolutional Sparse Representation. IEEE Signal Process. Lett..

[14] Liu Y., Chen X., Peng H., Wang Z.F. (2017). Multi-focus image fusion with a deep convolutional neural network. Inf. Fusion.

[15] Wei Q., Bioucas-Dias J., Dobigeon N., Tourneret J.Y. (2015). Hyperspectral and multispectral image fusion based on a sparse representation. IEEE Trans. Geosci. Remote Sens..

[16] Chen L., Li J.B., Chen C.L.P. (2013). Regional multifocus image fusion using sparse representation. Opt. Express.

[17] Tang H., Xiao B., Li W.S., Wang G.Y. (2018). Pixel convolutional neural network for multi-focus image fusion. Inf. Sci..

[18] Dian R.W., Li S.T., Fang L.Y., Wei Q. (2019). Multispectral and hyperspectral image fusion with spatial-spectral sparse representation. Inf. Fusion.

[19] Yin H.P., Li Y.X., Chai Y., Liu Z.D., Zhu Z.Q. (2016). A novel sparse-representation-based multi-focus image fusion approach. Neurocomputing.

[20] Xu M., Hu D.L., Luo F.L., Liu F.L., Wang S.Y., Wu W.W. (2020). Limited angle X ray CT reconstruction using image gradient *l*_0_ norm with dictionary learning. IEEE Trans. Radiat. Plasma Med. Sci..

[21] Zhang J., Zhao C., Zhao D.B., Gao W. (2014). Image compressive sensing recovery using adaptively learned sparsifying basis via *l*_0_ minimization. Signal Process..

[22] Cai T.T., Wang L. (2011). Orthogonal matching pursuit for sparse signal recovery with noise. IEEE Trans. Inf. Theory.

[23] Wang J., Kwon S., Shim B. (2012). Generalized orthogonal matching pursuit. IEEE Trans. Signal Process..

[24] Jeon M.J., Jeong Y.S. (2020). Compact and Accurate Scene Text Detector. Appl. Sci..

[25] Woźniak M., Silka J., Wieczorek M. (2021). Deep neural network correlation learning mechanism for CT brain tumor detection. Neural Comput. Appl..

[26] Vu T., Nguyen C.V., Pham T.X., Luu T.M., Yoo C.D. Fast and Efficient Image Quality Enhancement via Desubpixel Convolutional Neural Networks. Proceedings of the European Conference on Computer Vision (ECCV).

[27] Lytro Multi-Focus Dataset. https://mansournejati.ece.iut.ac.ir/content/lytro-multi-focus-dataset.

[28] Multi-Focus-Image-Fusion-Dataset. https://github.com/sametaymaz/Multi-focus-Image-Fusion-Dataset.

[29] Tsagaris V. (2009). Objective evaluation of color image fusion methods. Opt. Eng..

[30] Petrović V. (2007). Subjective tests for image fusion evaluation and objective metric validation. Inf. Fusion.

[31] Zhu Z.Q., Yin H.P., Chai Y., Li Y.X., Qi G.Q. (2018). A novel multi-modality image fusion method based on image decomposition and sparse representation. Inf. Sci..

[32] Chen Y., Blum R.S. (2009). A new automated quality assessment algorithm for image fusion. Image Vis. Comput..

[33] Xydeas C.S., Petrovic V.S. (2004). Objective image fusion performance measure. Electron. Lett..

[34] Zhao J., Laganiere R., Liu Z. (2007). Performance assessment of combinative pixel-level image fusion based on an absolute feature measurement. Int. J. Innov. Comput..

[35] Yang C., Zhang J.Q., Wang X.R., Liu X. (2008). A novel similarity based quality metric for image fusion. Inf. Fusion.

[36] Liu Z., Blasch E., Xue Z., Zhao J., Laganiere R., Wu W. (2011). Objective assessment of multiresolution image fusion algorithms for context enhancement in night vision: A comparative study. IEEE Trans. Pattern Anal. Mach. Intell..

[37] Zong J.J., Qiu T.S. (2017). Medical image fusion based on sparse representation of classified image patches. Biomed. Signal Process. Control.

[38] Liu Y., Liu S., Wang Z. (2017). A general framework for image fusion based on multi-scale transform and sparse representation. Inf. Fusion.

